# A Single Vaccination of IBDV Subviral Particles Generated by *Kluyveromyces marxianus* Efficiently Protects Chickens against Novel Variant and Classical IBDV Strains

**DOI:** 10.3390/vaccines9121443

**Published:** 2021-12-07

**Authors:** Deqiang Yang, Lixia Zhang, Jinkun Duan, Qiang Huang, Yao Yu, Jungang Zhou, Hong Lu

**Affiliations:** 1 State Key Laboratory of Genetic Engineering, School of Life Sciences, Fudan University, 2005 Songhu Road, Shanghai 200438, China; 18110700060@fudan.edu.cn (D.Y.); huangqiang@fudan.edu.cn (Q.H.); yaoyu@fudan.edu.cn (Y.Y.); 2 Shanghai Engineering Research Center of Industrial Microorganisms, 2005 Songhu Road, Shanghai 200438, China; 3 Tianjin Ruipu Biotechnology Co. Ltd., Tianjin 300350, China; lixiazhang@ringpu.com (L.Z.); jkduan@ringpu.com (J.D.); 4 Shanghai Collaborative Innovation Center for Biomanufacturing (SCICB), East China University of Science and Technology, 130 Meilong Road, Shanghai 200237, China

**Keywords:** novel variant IBDV, *Kluyveromyces marxianus*, subviral particles, vaccine, protection

## Abstract

Infectious bursal disease (IBD), caused by the infectious bursal disease virus (IBDV), is a highly contagious and immunosuppressive disease in chickens worldwide. The novel variant IBDV (nvIBDV) has been emerging in Chinese chicken farms since 2017, but there are no available vaccines that can provide effective protection. Herein, the capsid protein VP2 from nvIBDV strain FJ-18 was expressed in *Kluyveromyces marxianus* with the aim to produce nvIBDV subviral particles (SVPs). Two recombinant strains constructed for expression of nvIBDV VP2 (nvVP2) and His-tagged VP2 (nvHVP2) formed two types of nvIBDV subviral particles (SVPs), namely nvVP2-SVPs and nvHVP2-SVPs. TEM scans showed that both SVPs were about 25 nm in diameter, but there was a large portion of nvVP2-SVPs showing non-spherical particles. Molecular dynamics simulations indicate that an N-terminal His tag strengthened the interaction of the nvHVP2 monomer and contributed to the assembly of SVPs. Vaccination of chicks with the nvHVP2-SVPs provided 100% protection against novel variant IBDV infection when challenged with the FJ-18 strain, as well as the classical strain BC6/85. By contrast, vaccination with the nvVP2-SVPs only provided 60% protection against their parent FJ-18 strain, suggesting that the stable conformation of subviral particles posed a great impact on their protective efficacy. Our results showed that the nvHVP2-SVPs produced by the recombinant *K. marxianus* strain is an ideal vaccine candidate for IBDV eradication.

## 1. Introduction

Infectious bursal disease is a highly contagious infectious disease caused by the infectious bursal disease virus that leads to severe immunosuppression in chicks and causes huge economic losses to the poultry industry worldwide [1]. Taxonomically, there are two IBDV serotypes, serotype I and serotype II, but only the serotype I IBDV is pathogenic [2]. In 1957, IBDV first emerged in Gumboro town, Delaware, USA, and later evolved an antigenic variant strain and a very virulent strain. Accordingly, they were designated as classical IBDV (cIBDV), variant IBDV (vIBDV), and very virulent IBDV (vvIBDV), respectively, based on the antigenicity and virulence [3,4,5]. Since 2017, a novel variant IBDV (nvIBDV) with a distinct antigenicity from the earlier vIBDV, which gave rise to severe immunosuppression in chicken flocks and that is often associated with secondary infections of virus and pathogenic bacteria, has been emerging in many provinces in China and has brought about great economic losses [6,7,8]. Recently, this nvIBDV has also been identified in Japan [9] and South Korea [10]. The available commercial vaccines cannot provide complete protection against the nvIBDV infection, and this may be the main reason why nvIBDV spreads so fast among immunized flocks [7].

In addition to strict disinfection measures, prevention and control of IBD mainly relies on vaccination. Current commercial vaccines for IBD are mainly live-attenuated vaccines, inactivated whole-virus vaccines, and genetic engineering vaccines. Live-attenuated IBD vaccines can replicate in the bursa and induce humoral and cellular immunity simultaneously [11]. However, large-scale use of live-attenuated vaccines will cause genetic reassortment of vaccine strains and field strains, which accelerates the variation of IBDV [12,13,14,15]. On the other hand, those live-attenuated vaccines with intermediate virulence bring the risk of bursa atrophy and immunosuppression in the chicks [16]. As for inactivated whole-virus vaccines, they require repeated immunizations, which greatly increases the cost even though they are a little safer [17]. Subunit vaccines, especially virus-like particles, have shown great advantages in clinical applications. First, subunit vaccines are recombinant proteins without any nucleic acid of virus so they have a high bio-safety level. Secondly, the antigen protein of this vaccine can be produced by microbial cell factories with few expensive raw materials, greatly reducing the production cost. In addition, inoculation with subunit vaccines can easily achieve the differentiation of infected from vaccinated animals (DIVA), which is conducive to the eradication of IBDV [18].

*Kluyveromyces marxianus* (*K*. *marxianus*) is one of the fastest-growing eukaryotic organisms that has been recognized as GRAS by the FDA, as well as QPS by the EFSA [19]. It is also a promising cell factory for the production of virus-like particles (VLPs) [20,21]. In this study, we used *K. marxianus* to express nvIBDV VP2 protein with or without an N-terminal His tag. The yields of the two recombinant proteins reached about 1.06 g/L and 1.89 g/L, respectively, after 48 h fermentation. Both the nvVP2 and His-tagged nvVP2 proteins were self-assembled into SVPs with 25 nm in diameter, but some SVPs formed by the nvVP2 protein were non-spherical in conformation. Molecular dynamics simulations revealed that an N-terminal His tag strengthened the interaction of the nvHVP2 monomer, leading to a more stable structure than the nvVP2 pentamer. Vaccination with the nvHVP2-SVPs completely protected chicks against the infection of the novel variant and classical IBDV strains, while they were offered only 60% protection when vaccinated with the nvVP2-SVPs against novel variant IBDV strain FJ-18. This result indicates that the more stable structure of nvHVP2-SVPs induces a stronger immune response compared with the nvVP2-SVPs.

## 2. Materials and Methods

### 2.1. Plasmids, Yeast Strains and Viruses

The pUKDN115 plasmid vector and *K. marxianus* strain T1 *atg1*Δ were constructed previously [22] and stored in our laboratory. The nvIBDV strain FJ-18 and the cIBDV strain BC6/85 were kindly provided by Tianjin Ruipu Biotechnology Co. Ltd. (Ruipu, Tianjin, China).

The GenBank accession numbers of IBDV representative strains used in the sequence alignment are listed as follows: FJ-18 (OK167034), SHG4 (MH879084), SHG27 (MH879098), SHG311 (MH879123), Variant E (D10065), GLS (AY368653), 9109 (AY012683), HLJ0504 (GQ451330), HK46 (AJ878900), UK661 (NC004178), CJ801 (AF416621), and STC (AY819701).

### 2.2. Construction of the Plasmids for Expression of nvVP2 and nvHVP2 Proteins in K. marxianus

Total RNA was extracted from the bursa of Fabricius (BF), which was infected by the nvIBDV FJ-18 strain using RNAeasy™ Viral RNA Isolation Kit with Spin Column (Beyotime, Shanghai, China). The extracted RNA was then subjected to reverse transcription using PrimeScript™ RT reagent Kit (TaKaRa, China) following the manufacturer’s instruction. The VP2 and HVP2 genes were amplified by PCR using the reversely transcripted cDNA with the primer pairs VP2115F (5′-TTTTTTTGTT AGATCCGCGG ATGACAAACC TGCAAGATCA AACCC-3′)/VP2115R (5′-AGCTTGCGGC CTTAACTAGT TCACCTTAGG GCCCGAATTA TATCTTTG-3′) and HVP2115F (5′-TTTTTTTGTT AGATCCGCGG ATGCATCATC ACCATCACCA TACAAACCTG CAAGATCAAA CCC-3′)/HVP2115R (5′-AGCTTGCGGC CTTAACTAGT TCACCTTAGG GCCCGAATTA TATCTTTG-3′), respectively. Then, two expression plasmids, pUKDN115/VP2 and pUKDN115/HVP2, were constructed to express the nvVP2 and nvHVP2 proteins in *K. marxianus*, as described previously [20].

### 2.3. Transformations of the Expression Plasmids into K. marxianus

The two plasmids were transformed into the uracil auxotrophic *K. marxianus* strain T1 *atg1*Δ by the LiAc/carrier ssDNA/PEG method according to the previous research [23]. Due to the complement *URA3* gene in the pUKDN115 plasmid, transformants were selected on SD plates consisting of 10 g/L glucose, 6.7 g/L yeast nitrogen base, and agar 20 g/L).

### 2.4. SDS-PAGE and Western Blot Assays

The recombinant strains transformed with the plasmid pUKDN115/HVP2 and pUKDN115/VP2, respectively, grew in YG mediums (2% yeast extract, 4% glucose) at 30 °C, 220 rpm for 72 h. Yeast cells were harvested by centrifugation at 5000 rpm for 10 min and then suspended in PBS buffer (137 mM NaCl, 2.7 mM KCl, 10 mM Na_2_HPO_4_, 1.8 mM KH_2_PO_4_, pH 7.4). Cell disruptions were performed using a JN High Pressure Homogenizer (JNBIO, Guangzhou, China) under a condition of 1500 bar, 4 °C. Cell lysates were subjected to SDS-PAGE and Western blot assays using a chicken anti-IBDV serum with a dilution of 1: 1000 (Code Z33, China Institute of Veterinary Drugs Control, China) and a goat anti-chicken IgY secondary antibody HRP with a dilution of 1: 5000 (Code A16054, ThermoFisher Scientific, Waltham, MA, USA). The cell lysate of T1 *atg1*Δ transformed with pUKDN115 plasmid was used as negative control.

### 2.5. Agarose Gel Precipitation (AGP) Analysis

For AGP analysis, cell lysates were centrifuged at 10,000 rpm for 10 min, and the supernatants were serially diluted 1:16 to 1:256 with 0.9% NaCl solution, and 25 μL of diluted samples were subsequently added into the circumjacent wells of agarose plates. A total of 25 μL PBS buffer was used as negative control. An equal volume of reference IBDV antiserum purchased from China Institute of Veterinary Drugs Control was added into the central well. The plates were incubated at 37 °C for 48 h. The highest dilution of cell lysate at which the immunoprecipitation is still formed with the serum was designated as the AGP titer.

### 2.6. High-Density Fermentation of the Recombinant K. marxianus Strains

Fermenters (5 L, BXBIO, Shanghai, China) with 1.5 L mineral medium were sterilized at 110 °C for 15 min. Inoculum seeds were prepared by culture of the recombinant strains in 150 mL sterilized seed cultures (6.7 g/L yeast nitrogen base, 20 g/L glucose). After incubation at 30 °C, 220 rpm for 18 h, inoculum seeds were transferred into the 5 L fermenters. During fermentation, the dissolved oxygen was maintained within the range of 5–20%, and the temperature was controlled at 30 °C. The pH was automatically controlled at 5.5 with ammonium hydroxide. The composition of the mineral medium was prepared according to Hensing et al. [24], except for glucose as the carbon resource.

### 2.7. Quantification of the VP2 Proteins in K. marxianus after 48 h Fermentation

For quantification of the VP2 proteins, the cell lysates harvested from high-density fermentations at 48 h were diluted 1:10 with PBS buffer pH 7.4 and were subjected to SDS-PAGE analyses with different concentrations of bovine serum albumin (BSA) as standard. The grayscales of the VP2 proteins, as well as the different concentrations of BSA, in images of the SDS-PAGE gels were quantified with GenoSens Analysis software (Clinx Science, Shanghai, China). The yields of nvHVP2 and nvVP2 proteins were calculated according to the regression curve of BSA.

### 2.8. SE-HPLC Detection and Transmission Electron Microscopy

SE-HPLC detections of the cell lysate supernatants were performed as described previously [20]. The nvIBDV SVPs images were acquired by negative staining of the 14 min elutions in SE-HPLC detections with 2% (*w/v*) aqueous uranyl acetate, followed by observation under the Tecnai G^2^ F20 S-Twin microscope at an accelerating voltage of 200 kV.

### 2.9. Animal Experiments

Specific-pathogen-free (SPF) chickens were purchased from Beijing Boehringer Ingelheim Vital Biotechnology Co., Ltd. and were fed in the negative-pressure-filtered air isolators. All animal experiments were approved by the Ethics Committee of Tianjin Ruipu Biotechnology Co. Ltd. (protocol code SYXK (Jinbin) 2021-0004).

In experiment 1, thirty 3-week-old SPF chickens were randomly divided into four groups, the KM-HVP2 (*n* = 10), the KM-VP2 (*n* = 5), the challenge control (CC, *n* = 10), and the negative control (NC, *n* = 5). The purified nvHVP2-SVPs and nvVP2-SVPs were diluted to 1:32 AGP titer and emulsified 1:2 with oil adjuvant. In the KM-HVP2 and the KM-VP2 groups, chickens were subcutaneously immunized with 0.2 mL emulsified nvHVP2-SVPs and nvVP2-SVPs, respectively. Chickens in the CC and the NC groups were injected with 0.2 mL PBS. Blood samples were collected at 14 days post immunization (dpi). At 21 dpi, all the chickens except the NC group were orally challenged with nvIBDV strain FJ-18 at a dose of 100 BID_50_ (50% bursa infectious dose). At 4 days post challenge (dpc), all chickens were euthanized and necropsied. The bursa, spleen, and the whole body for all chickens were weighed to calculate the bursa/body weight indexes (BBIX) (BBIX = (bursa: body weight ratios)/(mean value of bursa: body weight ratios in the NC group)) and spleen/body weight indexes (PBIX) (PBIX = (spleen: body weight ratios)/(mean value of spleen: body weight ratios in the NC group)). A bursa with a BBIX less than 0.70 was considered as atrophied. A bursa/spleen with a BBIX/PBIX of more than 1.50 was considered as edema. Other gross lesions in the bursa, such as hemorrhage, turning yellow, or jelly-like exudation, were also recorded.

In experiment 2, twenty 3-week-old SPF chickens were randomly divided into three groups, the KM-HVP2 (*n* = 5), the challenge control (CC, *n* = 10), and the negative control (NC, *n* = 5). Chickens in the KM-HVP2 group were immunized with 0.2 mL nvHVP2-SVPs vaccine, the same as above. Chickens in the CC and the NC groups were administered with 0.2 mL PBS. At 21 dpi, chickens in both the KM-VP2 group and the CC group were challenged orally with 100 BID_50_ of the classical IBDV strain BC6/85. Similarly, the indexes of BBIX and PBIX were determined as described above.

### 2.10. Enzyme-Linked Immunosorbent Assay

The 96-well plates (One Riverfront Plaza Corning, Corning, NY, USA) were coated with 100 ng per well of purified nvIBDV VP2 protein expressed by *Pichia pastoris* (*P*. *pastoris*) (Ruipu, Tianjin, China) in the coating buffer (15 mM Na_2_CO_3_, 35 mM NaHCO_3_, pH 9.6) at 4 °C overnight and then blocked by 5% non-fat milk at room temperature for 1 h [25]. After that, 100 μL of 1:500 diluted chicken sera was added to each well of the coated plates. The plates were incubated at 37 °C for 30 min, followed by washing with PBST three times. The goat anti-chicken IgY secondary antibody HRP (Invitrogen, Corning, NY, USA) diluted with PBST (137 mM NaCl, 2.7 mM KCl, 10 mM Na_2_HPO_4_, 1.8 mM KH_2_PO_4_, 0.05% Tween-20, pH 7.4) was added, and the plates were incubated at 37 °C for another 30 min. After washing three times with PBST, the plates were visualized by adding 100 μL 3,3′,5,5′-tetramethylbenzidine solution (Tiangen, Beijing) and incubated for 10 min at room temperature. Reactions were terminated by adding 50 μL of 2 M sulfuric acid. Absorbance at 450 nm was recorded using an Eon™ High Performance Microplate Spectrophotometer (BioTek, Shoreline, WA, USA). The S/P value was calculated by the following formula: (S/P value = (sample value − mean value of NC)/(mean value of PC − mean value of NC)). A sera sample with an S/P value > 0.2 was considered positive.

### 2.11. Real-Time Quantitative PCR Analysis

Real-time PCR analysis was conducted to determine the IBDV viral load in the bursa of the challenged chickens. Viral RNA was extracted from chicken bursa using RNAeasy™ Viral RNA Isolation Kit (Beyotime, Shanghai, China) according to the manufacturer’s instruction. Reverse transcriptions were conducted using a PrimeScript™ RT reagent Kit (TaKaRa, Japan). Quantification of IBDV virus titers in bursas was conducted by qPCR with the primers IBDV-qF (5′-ACTCCCTGGT GGCGTTTATG-3′) and IBDV-qR (5′- ACCCCTTCCC CTACTAGGAC-3′) using cDNA as the templates and the plasmid pUKDN115/VP2 as the reference.

### 2.12. Statistical Analysis

Statistical analysis was performed with Graphpad Prism 6 software. An unpaired *t*-test was applied to evaluate the statistical differences between groups. A *p*-value < 0.05 was considered to be statistically significant.

## 3. Results

### 3.1. Expression and Identification of the nvVP2 and nvHVP2 Proteins in K. marxianus

**Figure 1:**
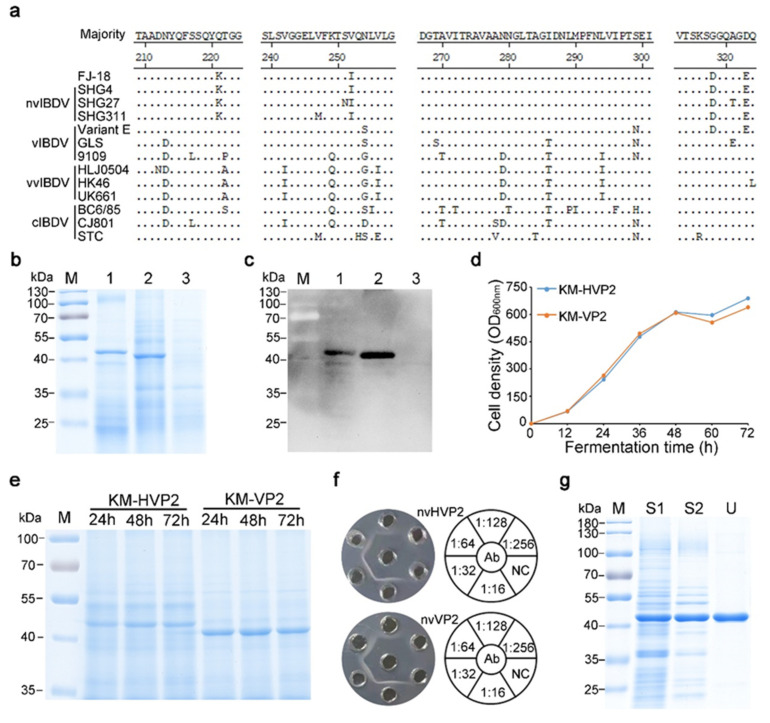
Expression and identification of the nvHVP2 and nvVP2 proteins in *K. marxianus*. (**a**) Alignment of the amino acid sequences for VP2 high variable region (aa 206–350) from different IBDV strains. SDS-PAGE (**b**) and Western blot (**c**) analyses of soluble cell lysates of KM-HVP2 and KM-VP2 strains cultured in YG medium for 72 h. Lane M: Prestained Protein Ladder; Lane 1: Soluble cell lysates of the KM-HVP2 strain. Lane 2: Soluble cell lysates of the KM-VP2 strain. Lane 3: Soluble cell lysates of T1 *atg1*Δ transformed with pUKDN115. (**d**) Growth curves of the KM-HVP2 and KM-VP2 strains in 5 L fermenters. (**e**) SDS-PAGE analysis of the nvHVP2 and nvVP2 proteins in tenfold diluted cell lysates at indicated fermentation times. (**f**) The AGP titers for the cell lysates of the KM-HVP2 and KM-VP2 strains after 48 h fermentation. Ab: Reference IBDV antiserum. (**g**) SDS-PAGE of the nvHVP2 proteins purified by the pH adjustment coupled with ultrafiltration. The KM-HVP2 cells were disrupted in PBS buffer pH 7.4 and were then adjusted to pH 4.2 using acetic acid. After centrifugation at 12,000 rpm for 15 min, supernatant was ultrafiltrated on a 750 kDa column under a tangential flow filtration. Lane S1: The supernatant of KM-HVP2 cell lysate before pH adjustment; Lane S2: the pH adjusted supernatant; Lane U: the fraction after ultrafiltration.

In 2018, the IBDV FJ-18 strain was isolated in a chicken farm in Fujian, China. The hypervariable region of the FJ-18 VP2 protein (aa 206–350) was aligned with the same region of other representative strains belonging to the nvIBDV, vIBDV, vvIBDV, and cIBDV. The result showed that the homologies of FJ-18 strain to other nvIBDV isolates were 98.6–100%. As a contrast, homologies to the representative strains of other genotypes, such as vIBDV, vvIBDV, and cIBDV, were 90.3–97.2%, 90.3–91.0%, and 88.3–91.7%, respectively (Figure 1a). Accordingly, the FJ-18 strain in this study is classified into the nvIBDV genotype.

The VP2 gene of the FJ-18 strain, or with an N-terminal His-tag sequence, was amplified and cloned into the pUKDN115 vector separately, obtaining the recombinant plasmids pUKDN115/HVP2 and pUKDN115/VP2. Then two recombinant *K. marxianus* strains, KM-HVP2 and KM-VP2, were constructed by transformation of the above two plasmids respectively. After cultivation in YG medium for 72 h, yeast cells were collected and disrupted in PBS buffer (pH 7.4), and the supernatants were subjected to SDS-PAGE and Western blot analysis. From SDS-PAGE, specific bands corresponding to the nvHVP2 and nvVP2 protein molecular weights were observed in the supernatants of KM-HVP2 and KM-VP2 cell lysates (Figure 1b), which were further confirmed by Western blot using chicken anti-IBDV serum (Figure 1c).

Subsequently, scale-up fermentations were performed through a fed-batch method in 5 L bioreactors. After 72 h of fermentation, the cell densities (OD_600nm_) of the two strains reached 689 and 640, respectively (Figure 1d). Cells collected every 24 h were analyzed for the expression level of the VP2 proteins. As shown in Figure 1e, the highest productions for both nvHVP2 and nvVP2 were found at 48 h; however, prolonging the fermentation time decreased the yields of both proteins, even though the cell densities increased until 72 h. The yields of nvHVP2 and nvVP2 proteins reached 1.06 g/L and 1.89 g/L, respectively. In the AGP tests, both soluble extracts of cell lysates formed immunoprecipitation with the reference IBDV antiserum, and the AGP titers were both 1:128, irrespective of the different concentrations of the two proteins (Figure 1f).

When the pH of cell lysate lowered to 4.2, impurities in the soluble fractions declined significantly but, interestingly, there was no obvious loss of the nvHVP2 protein in SDS-PAGE analysis (Figure 1g). This facile treatment provides a cost-effective way to efficiently purify the nvHVP2 protein from yeast cell lysates. To remove small molecules, the supernatant of the pH-adjusted cell lysate was transferred into a 750 kDa column to conduct a tangential flow filtration. After ultrafiltration, the nvHVP2 protein with high purity was obtained (Figure 1g). Similarly, high purity of the nvVP2 protein could also be achieved by the same treatments.

### 3.2. The nvVP2 and nvHVP2 Proteins Expressed in K. marxianus Self-Assemble to Form SVPs

**Figure 2:**
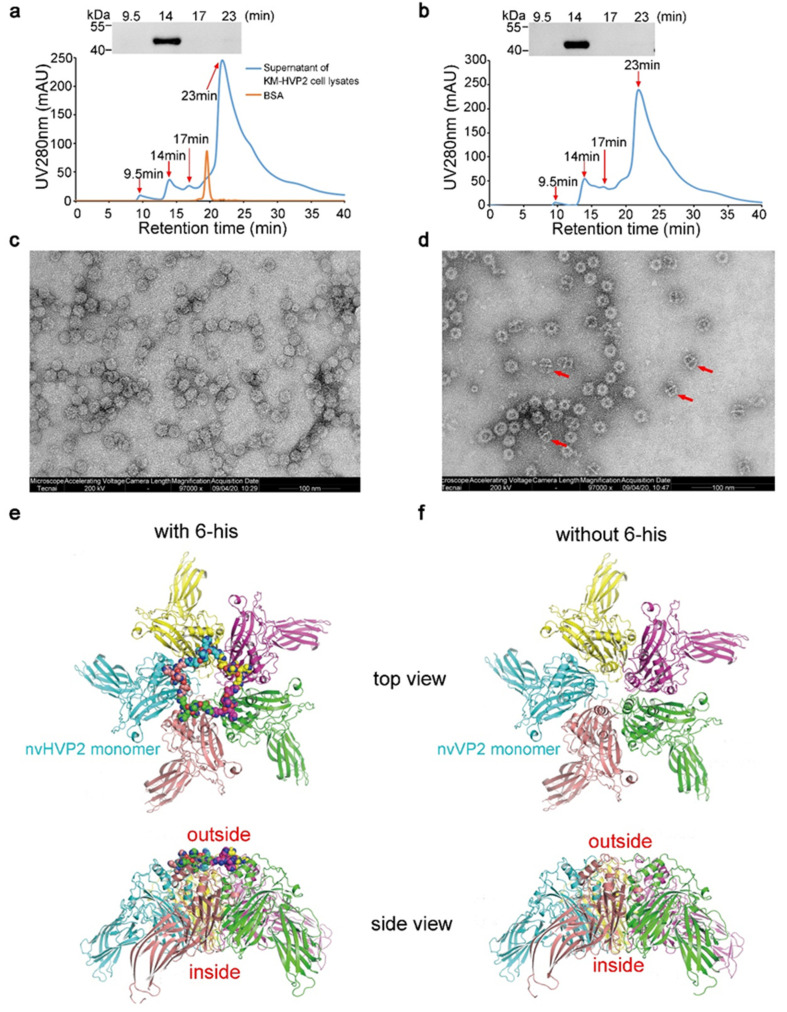
Identifications and MD simulations of the nvHVP2 and nvVP2 assembly. SE-HPLC and Western blot analyses of the nvHVP2-SVPs (**a**) and nvVP2-SVPs (**b**) in cell lysates. TEM scans of the nvHVP2-SVPs (**c**) and nvVP2-SVPs (**d**) that were collected at 14 min in SE-HPLC. Bars, 100 nm. MD simulations of the nvHVP2 pentamer (**e**) and nvVP2 pentamer (**f**). All pictures were exported by the PyMOL software.

To detect whether the nvVP2 and nvHVP2 proteins assemble into SVPs, the supernatants of cell lysates were fractionated with SE-HPLC. As an internal reference, bovine serum albumin (BSA), a protein with a molecular weight of 67 kDa, was eluted at 19 min in SE-HPLC. In the supernatants of KM-HVP2 and KM-VP2 cell lysates, four elution peaks were detected, of which the retention times were 9.5, 14, 17, and 23 min, respectively. Meanwhile, the four major peaks were collected and analyzed by Western blot. As shown in Figure 2a, only the fractions eluted at 14 min could be detected by the chicken anti-IBDV serum, indicating this fraction is the elution of nvHVP2 or nvVP2 protein. Compared to BSA, the predicted molecular weights of the nvHVP2 and nvVP2 proteins (46 and 45 kDa respectively) are higher, but their retention times were earlier in SE-HPLC. This suggested that the nvHVP2 and nvVP2 proteins expressed in *K. marxianus* might self-assemble into polymers with nearly 100% of the assembly efficiency.

A TEM scan showed that the nvHVP2 protein assembled into uniform spherical SVPs with a diameter of about 25 nm (Figure 2c), while a considerable proportion of nvVP2 protein aggregated into a non-spherical form of polymers (Figure 2d). To find out the contribution of the His tag to the stability of nvHVP2 SVPs, molecular dynamics (MD) simulation was employed to predict the 3D structures of two proteins (nvHVP2 and nvVP2) via the Swiss-Model server (https://swissmodel.expasy.org/, 21 August 2021). The used structural template of the monomer was that from the IBDV capsid structure (PDB ID 2DF7). Then, two capsid pentamers were constructed with the obtained monomer structures according to PDB 2DF7. Surprisingly, we found that the six N-terminal histidines of nvHVP2 interacted with each other at the pentamer interface (the sphere models in Figure 2e). In contrast, there are no such monomer interactions in the pentamer of nvVP2 (Figure 2f). As seen in Figure 2e, the N-terminal histidines of the nvHVP2 monomers form an additional ring-like structure at the center of the pentamer. It is likely that such additional ring-like structures could further stabilize the structure of the nvHVP2 capsid. However, to confirm this, a high-resolution structural determination of the nvHVP2 capsid is needed and will be presented in the future study.

### 3.3. Immunoprotections of the nvHVP2-SVPs and nvVP2-SVPs against IBDV Infection

**Figure 3:**
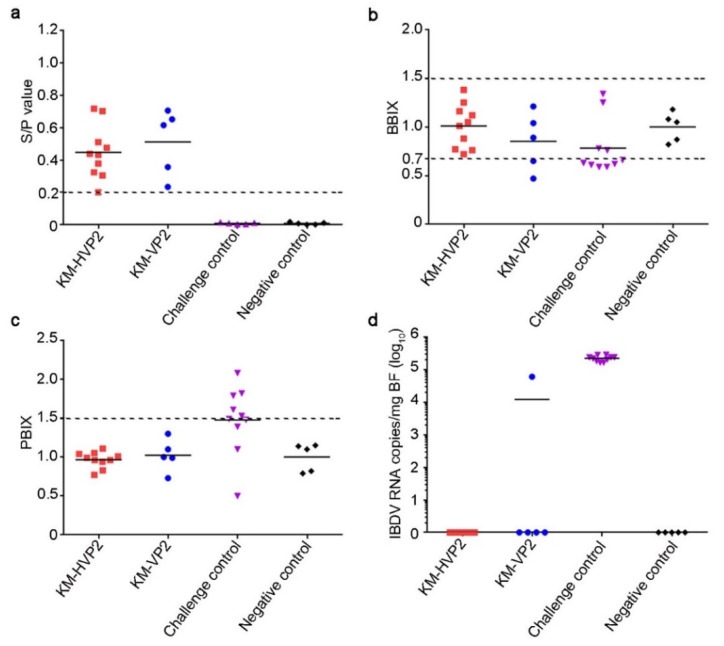
Immunoprotections of the nvIBDV SVPs vaccines against nvIBDV FJ-18 strain. (**a**) The specific anti-nvVP2 IgY antibodies in chicken sera of the KM-HVP2, KM-VP2 group, challenge control, and negative control groups. A serum sample with an S/P value > 0.2 was considered to be seropositive. (**b**) BBIX in each group. A bursa with a BBIX of less than 0.7 was considered as atrophied, and a BBIX of more than 1.5 was considered as edema. (**c**) PBIX in each group. Spleen with a PBIX of more than 1.5 was considered as edema. (**d**) The nvIBDV virus copy number in bursa in each group.

Three-week-old SPF chicks were immunized with nvHVP2-SVPs and nvVP2-SVPs vaccines, and protections were evaluated by challenging with the nvIBDV FJ-18 strain. Two weeks post immunization, chicken sera were collected for the detections of the antibody by ELISA. As a result, the titers of nvIBDV VP2 antibodies in all chicken sera from both the KM-HVP2 and the KM-VP2 groups were higher than the value of seroconversion. Meanwhile, no chicken sera from the non-immunized groups were seropositive in ELISA detection (Figure 3a). After 4 dpc with the IBDV FJ-18 strain, BBIX suggested that the bursas of all birds in the KM-HVP2 group were normal in size, while in the KM-VP2 group, two bursas were atrophied (Figure 3b and Table 1). The result of PBIX also showed the spleen of all immunized chicks had no significant difference in size compared with the chicks in the NC group, but the PBIX of the CC group was significantly higher than that of the NC group (Figure 3c). Other gross lesions, such as turning yellow, hemorrhage, and inflammation exudation, were found in bursas of all chickens in the CC group, while no such lesions were observed in other groups (Table 1). However, due to concern that low viral load in the tissue might cause unobvious injuries, histological analyses were not carried out, but the IBDV viral loads in the bursas were qualified by qPCR. Just as in the NC group, no IBDV virus was detected in bursas of chickens from the KM-HVP2 group. However, there was one chicken in the KM-VP2 group, as well as chickens in the whole CC group, infected with high copies of the FJ-18 virus (Figure 3d and Table 1). In summary, the nvHVP2-SVPs vaccine provided complete protection against the nvIBDV FJ-18 infection, while the protective efficacy of the nvVP2-SVPs vaccine was only 60%.

**Figure 4:**
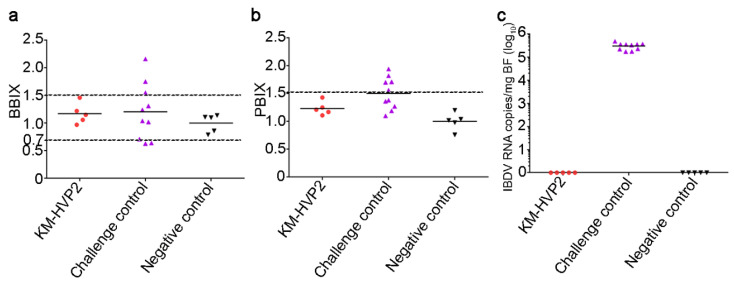
Immunoprotections of the nvHVP2-SVPs vaccine against IBDV BC6/85 strain infection. The BBIX (**a**) and PBIX (**b**), as well as the titers of BC6/85 virus in bursas (**c**) in each group, were analyzed to assess the immunoprotections of the nvHVP2-SVPs vaccine against classical IBDV strain BC6/85 infection.

In order to evaluate the cross-protection efficacy from the classical IBDV strain infection, 3-week-old SPF chickens were immunized with nvHVP2-SVPs vaccine and challenged at 21 dpi with the BC6/85 strain. Despite no chickens dying at 4 dpc, BBIX showed that five bursas in the CC group had atrophy or edema, and PBIX revealed that half of the chickens had edema in the spleen (Figure 4a,b). However, no chickens in the KM-HVP2 group showed abnormalities either in the bursa or spleen, just as the animals in the NC group (Figure 4a,b). Quantifications of the viral loads in the bursal tissues showed that IBDV was undetectable in both the NC and the KM-HVP2 groups, but high titers of BC6/85 virus existed in the bursa of all chicks in the CC group that were not immunized with any IBDV vaccine and challenged (Figure 4c). Compared with the NC group, all chicks in the CC group showed significant pathological changes in BF, while chicks in the KM-HVP2 group showed no visible pathological changes in the bursa (Table 2). This result indicates that the nvHVP2-SVPs vaccine also has a complete cross-protection of chickens against the classical IBDV BC6/85 strain.

## 4. Discussion

Since 2017, nvIBDV has been emerging in chicken flocks from many provinces in China. It is reported that the antigenicity of nvIBDV has changed significantly compared with the previous genotype [6]. Unlike the high mortality of vvIBDV genotype, nvIBDV infection is not lethal to chicks, but it can cause severe immunosuppression, leading to subsequent infections in flocks [7]. It is deemed that nvIBDV has become a growing threat to the poultry industry. To develop a specific vaccine, in this study, the nvIBDV outer capsid protein VP2, or with an N-terminal His tag, was expressed by *K. marxianus*. The N-terminal His tag enhanced the stability of nvHVP2-SVPs, thus making the nvHVP2-SVPs vaccine more efficacious than the nvVP2-SVPs vaccine.

The VP2 protein is the only protein that formed the outer capsid of IBDV. As a scaffold protein, VP3 protein locates inside the IBDV capsid to form a double-layer structure. Therefore, the VP2 protein is the ideal candidate for the development of subunit IBDV vaccine. Maturation of the VP2 protein (441 aa) undergoes a series of hydrolyses from its precursor pVP2 (512 aa) [26]. As the recombinant expression of IBDV VP2 protein or some of its intermediates results in a tubular structure, the length of VP2 protein is very crucial for its spherical assembly. The amphipathic α-helix (α5 helix) located at 443–452 aa of VP2 protein has been proven to be a conformational switch for the VP2 protein polymorphism [27]. Interaction between the α5 helix and VP3 protein could help form the conformation of IBDV correctly, thus the first 452 aa of nvIBDV VP2 protein was used for recombinant expression in *K. marxianus*. A previous report revealed that the electrostatic interaction between the last five residues of the VP3 C-terminal and the α5 helix regulated the polymorphism of the VP2 protein [28]. The His tag has a similar charge distribution to the C-terminal residues of VP3, which may mimic the function of the VP3 C-terminus and promote the correct assembly of SVPs through interaction with the α5 helix [29]. Due to a decisive role in the protein assembly, the N-terminal His tag could be an effective means to enhance the immune efficacy of the VP2 protein. Inevitably, the nvHVP2-SVPs provided complete protection for chicks against nvIBDV FJ-18 strain infection, while nvVP2-SVPs without His tag had only 60% protection against the parent strain after immunization.

Given that multiple IBDV genotypes are epidemic in China simultaneously, cross-protection is a particularly important factor in the vaccine development [30]. A recent study showed that the recombinant nvIBDV VP2 (466 aa) protein expressed by *E. coli* provides 100% protection against both nvIBDV and vvIBDV genotypes [31]. The results are encouraging, but whether the nvIBDV vaccine can provide cross-protection against genotypes other than vvIBDV remains to be studied. Therefore, we conducted a challenge test with the cIBDV strain BC6/85, and the nvIBDV SVPs demonstrated a 100% cross-protection against classical IBDV. However, another study showed that commercial vvIBDV vaccines provide only 50% protection against nvIBDV [32]. It seems that broad cross-protection of nvIBDV vaccines is provided by its universal neutralizing epitopes [31]. Nevertheless, its cross-neutralizing activity was not high against vvIBDV genotypes after immunization by nvIBDV vaccine. Presumably, cellular immunity may play an important role in providing high cross-protection efficacy.

Development of a practical IBDV vaccine requires not only high protection efficacy but also a high yield to lower the production cost. IBDV SVPs have been expressed in various expression hosts, such as baculovirus–insect cell (B/IC), yeast, and bacteria [33,34,35]. The B/IC system is the most commonly used system for large-scale production of veterinary vaccines but the high cost of the time-consuming, costly culture medium, and the up- and downstream complexity are the main disadvantages for these systems in competition with other systems, and potential safety concerns are still a challenge [36,37,38]. *E. coli* is a well-established system for the high-level expression of heterologous proteins and offers ease of scale-up, making it an attractive host. However, it lacks a mammalian-like post-translational modification (PTM), which is very important for the immunogenicity of some SVPs/VLPs [36], and contains endotoxins, bacterial lipopolysaccharides that can cause severe side effects such as septic shock and tissue damage, that require an extra separation step to remove, thus increasing the complexity of purification processes and the final production cost [39]. As a unicellular eukaryotic microbe, yeast is considered as a particularly powerful platform for SVPs/VLPs production because it possesses PTM, grows cheaply and rapidly, and readily ferments in high cell density [40]. There are a growing number of vaccines, with more than 30 types that have been produced in three yeast species, including *Saccharomyces cerevisiae*, *P. pastoris, K. marxianus,* and *Hansenula polymorpha* [21,41]. More importantly, production of IBDV SVPs in yeast such as *K. marxianus* and *P. pastoris* reaches more than 1.0 g/L, which is significantly higher than in B/IC and *E*. *coli*, since the expression levels in these two hosts were lower than 100 mg/L with the maximum AGP titer of no more than 1:16 [34,35,42]. In yeast, however, particle-based vaccines are commonly expressed and assembled intracellularly; thus, a mechanical, chemical, or enzymatic method should be employed to disrupt the rigid wall of the yeast cells to release VLPs after cell harvesting, which makes the purification process more complex. Interestingly, this obstacle has been easily cleared by a pH adjustment in the case of the nvIBDV SVPs. Coupled with ultrafiltration, the purity of nvIBDV SVPs reached more than 70% (Figure 1g). The purification process established in this study provides a simple, cost-effective, and efficient way for large-scale production of IBDV SVPs by yeast.

## 5. Conclusions

In summary, the discrepancy in the conformations of two SVPs formed by nvIBDV VP2 protein with or without N-terminal His tag led to a different immunoprotection against nvIBDV infection. The nvIBDV SVPs formed by the nvHVP2 protein completely protected chickens against either the nvIBDV strain or the cIBDV strain through a single dose and achieved sterile immunity simultaneously. That it is high yield, low cost, and easy to use made nvHVP2-SVPs a promising vaccine candidate for IBDV eradication.

## Figures and Tables

**Table 1:** Protective properties of nvIBDV SVPs vaccines on bursa of Fabricius and spleen against nvIBDV strain FJ-18 infection.

Groups	Bursa Atrophy/Edema	Spleen Edema	Gross Lesion in Bursa	IBDV in Bursa
KM-HVP2	0/10	0/10	0/10	0/10
KM-VP2	2/5	0/5	0/5	1/5
Challenge control	6/10	5/10	10/10	10/10
Negative control	0/5	0/5	0/5	0/5

**Table 2:** Protective properties of nvHVP2-SVPs vaccine on bursa of Fabricius and spleen against cIBDV strain BC6/85 infection.

Groups	Bursa Atrophy/Edema	Spleen Edema	Gross Lesion in Bursa	IBDV in Bursa
KM-HVP2	0/5	0/5	0/5	0/5
Challenge control	5/10	5/10	10/10	10/10
Negative control	0/5	0/5	0/5	0/5

## Data Availability

Data can be requested by writing to the author.
